# Analysis of risk factors for multidrug-resistant bacterial infections in intensive care units and application of a nomogram prediction model: Erratum

**DOI:** 10.1097/MD.0000000000050459

**Published:** 2026-09-04

**Authors:** Yi Fu, Xing-ran Zhao, Jian-jun Luo, Fang Li, Zhi-hong Zhang, Ru-yu Li, Yu-xue Liu

In the article “Analysis of risk factors for multidrug-resistant bacterial infections in intensive care units and application of a nomogram prediction model,”^[1]^ which appears in Volume 105, Issue 29 of *Medicine*, the first row of Table 1 was incorrectly presented as “Sex, n (%) with total value of 413.” Table 1 has now been corrected online as “Female, n (%) with total value of 503.”

Additionally, the published article incorrectly states the study period as "January 2020 to December 2025." Therefore, all occurrences of “January 2020 to December 2025” in the published article have been corrected to “January 2021 to December 2025.”

**Table 1:** Baseline characteristics of ICU patients with and without multidrug-resistant (MDR) bacterial infection at Leshan People’s Hospital, China, January 2021–December 2025 (n = 876).

Variable	Total (n = 876)	MDR (n = 161)	Non-MDR (n = 715)	*P*
Female, n (%)	503	73	430	.534
Age > 75 yr, n (%)	248	64	184	.005
ICU stay > 1 wk, n (%)	203	53	150	.002
Coma, n (%)	135	40	95	.006
Surgery, n (%)	317	53	264	.185
Blood transfusion, n (%)	246	54	192	.113
Hypertension, n (%)	357	74	283	.195
Diabetes, n (%)	297	58	239	.462
Cardiovascular/cerebrovascular disease, n (%)	413	86	327	.123
Multiple injuries, n (%)	298	56	242	.741
Pulmonary infection, n (%)	265	61	204	.026
Suctioning, n (%)	217	53	164	.019
Endotracheal intubation, n (%)	118	34	84	.005
Central venous catheterization, n (%)	527	120	407	.001
Drainage tube placement, n (%)	126	30	96	.122

ICU = intensive care unit.
